# Draft Genome Sequence of Biocontrol Agent *Pythium oligandrum* Strain Po37, an Oomycota

**DOI:** 10.1128/genomeA.00215-16

**Published:** 2016-04-14

**Authors:** Harald Berger, Amira Yacoub, Jonathan Gerbore, Damien Grizard, Patrice Rey, Angela Sessitsch, Stéphane Compant

**Affiliations:** a AIT Austrian Institute of Technology GmbH, Bioresources Unit, Health and Environment Department, Tulln, Austria; b Fungal Genetics and Genomics Unit, BOKU University and AIT Austrian Institute of Technology, Bioresources & Technologies Campus Tulln, Tulln/Donau, Austria; c Institut National de Recherche Agronomique, ISVV, UMR1065 Santé et Agroécologie du Vignoble (SAVE), Villenave d'Ornon, France; d Université de Bordeaux, Bordeaux Sciences Agro UMR1065 SAVE, ISVV, Villenave d'Ornon, France; e BIOVITIS, Saint-Étienne-de-Chomeil, France

## Abstract

The oomycota *Pythium oligandrum* Po37 is used as a biocontrol agent of plant diseases. Here, we present the first draft of the *P. oligandrum* Po37 genome sequence, which comprises 725 scaffolds with a total length of 35.9 Mb and 11,695 predicted protein-coding genes.

## GENOME ANNOUNCEMENT

*Pythium oligandrum* strain Po37 has been isolated from the grapevine rhizosphere (plants grown in stony soils in Bordeaux, France) (1). This oomycete is well known for its ability to control phytopathogens, mainly through the stimulation of plant defense systems (2). *P. oligandrum* is also known for its three elicitin-like proteins, the extracellular glycoprotein oligandrin and the 2 cell wall proteins (POD1 and POD2), which are able to induce plant resistance against pathogens. Strain Po37 has been reported to protect *Vitis vinifera* L. against a pathogen involved in grapevine trunk diseases, *Phaeomoniella chlamydospora*, through the induction of plant resistance (3). This biocontrol agent colonizes the grapevine rhizosphere and induces several genes involved in various pathways (i.e., pathogenesis-related [PR] proteins and phenylpropanoid pathways). The genome sequencing might give information to gain insight into the plant-microorganism relationship, the phylogenetic status, and the biotechnology relevance of this microorganism.

The strain was cultivated on potato dextrose agar (PDA), tip purified, and DNA was isolated using DNeasy plant minikit (Qiagen) for phylogenetic testing by internal transcribed spacer (ITS)-large subunit (LSU)-rRNA gene sequencing. Sequencing was performed by GATC Biotech AG (Konstanz, Germany) using an Illumina MiSeq personal sequencer in paired-end 250-bp mode (MiSeq reagent kits version 2). A total of 14.51 M reads were received, representing in total 7,255.1 M bases. A read quality check was performed using FastQC (http://www.bioinformatics.babraham.ac.uk/projects/fastqc/). Raw sequence reads were quality filtered using Trimmomatic (4) to finally obtain 2 × 14.2 M paired reads that were used for assembly. The de Bruijn graph-based assembler SPAdes (5) was used for the scaffold assembly after estimating the optimal k-mer lengths with KmerGenie (6). Seven hundred twenty-five scaffolds >2 kb were obtained, with a mean coverage of 130× and total length of 35.9 Mb. The quality of the genome assembly was assessed in QUAST (7) (*N*_50_, 119,987; *N*_75_, 69,828; *L*_50_, 92; and *L*_75_, 188). Sequencing completeness was estimated using BUSCO (8) based on a set of 1,438 common fungal genes, aka benchmarking universal single-copy orthologs (BUSCOs). Eight hundred twenty-two complete single-copy BUSCOs, 213 fragmented BUSCOs, and 87 duplicated BUSCOs were found, leading to missing 403 BUSCOs in *P. oligandrum* Po37. We also screened already published genome sequences of related oomycetes, *Pythium ultimum* DAOM BR144 (9) and *Phytophthora parasitica* INRA-310 (10), and found 433 and 423 BUSCOs missing, respectively. Therefore, the sequencing was considered completed.

BUSCO was also used to calculate a training set for the gene predictor Augustus (11), and this method predicted 11,695 genes, whose translated sequences were submitted to a BLASTp search against the nr database (NCBI) or CAZydb (12). The Carbohydrate-Active enZYmes (CAZy) Database search revealed 236 glycoside hydrolases defined as enzymes for hydrolysis and/or rearrangement of glycosidic bonds, 258 glycosyltransferases (formation of glycosidic bonds), 24 polysaccharide lyases (nonhydrolytic cleavage of glycosidic bonds), 111 carbohydrate esterases (hydrolysis of carbohydrate esters), 95 redox enzymes that act in conjunction with CAZymes, and 85 enzymes with carbohydrate-binding modules that act in adhesion to carbohydrates.

Among all predicted protein-coding genes, genes related to the elicitin-like proteins oligandrin and the cell wall proteins were found in the genome of strain Po37, explaining its ability to act as an inducer of plant systemic resistance.

The Po37 genome sequence will help in understanding the specific properties of this strain.

### Nucleotide sequence accession numbers.

This whole-genome shotgun project has been deposited at DDBJ/ENA/GenBank under the accession no. LSAJ00000000. The version described in this publication is version LSAJ01000000.
